# Using informant discrepancies in report of parent–adolescent conflict to predict hopelessness in adolescent depression

**DOI:** 10.1177/1359104520969761

**Published:** 2020-11-05

**Authors:** Erling W Rognli, Marianne Aalberg, Nikolai Olavi Czajkowski

**Affiliations:** 1 Department of Psychology, University of Oslo, Norway; 2 Department of Child and Adolescent Mental Health Services, Akershus University Hospital, Lørenskog, Norway; 3 Division of Mental Health Services, Akershus University Hospital, Lørenskog, Norway; 4 PROMENTA Research Center, Department of Psychology, University of Oslo, Norway; 5 Division of Mental Health, Norwegian Institute of Public Health, Oslo, Norway

**Keywords:** Hopelessness, adolescent depression, informant discrepancies, latent difference scores, parent–adolescent conflict, Bayesian data analysis

## Abstract

Hopelessness is an important symptom of adolescent depression, being associated with both risk of suicide and poor treatment response, but predictors of hopelessness are understudied. Conflict with parents is common in adolescent depression, but parents and adolescents often disagree when reporting conflict severity. Discrepancy in reporting may be an indicator of the parent–adolescent dyad lacking a shared representation of the state of their relationship. This could make conflicts seem unresolvable to the adolescent, leading to expectations of persistent stress and lack of support, increasing hopelessness. This study employed latent difference scores, ordinal regression and cross-validation to evaluate the hypothesis that discrepancy in report of parent–adolescent conflict would predict hopelessness among depressed adolescents. Parents reporting less conflict than the adolescent was associated with increased adolescent hopelessness, giving preliminary support to the hypothesis.

## Introduction

Hopelessness is a state where a person expects negative events to occur, feels important goals cannot be achieved and that they are powerless to improve their future, either by their own agency or by the help of others (Marchetti, 2018). As a symptom of adolescent depression, hopelessness is particularly important, as it is a well-established predictor of adolescent suicidal behaviour (Wolfe et al., 2019), and has been shown to predict poor treatment response (Emslie et al., 2011). There is also some evidence that hopelessness is implicated in the development of depression (Alford et al., 1995).

### Predictors of hopelessness

Not all depressed adolescents experience hopelessness (Yorbik et al., 2004), and very few studies has previously investigated predictors of hopelessness in clinical samples. Kashani et al. (1994) found a lack of supportive relationships to predict hopelessness in a sample of preadolescent inpatients with various diagnoses. Becker-Weidman et al. (2009) modelled both cognitive and socio-environmental variables as possible predictors of hopelessness in a large clinical sample of adolescents with a diagnosed depressive disorder. They found cognitive distortions in view of self and the world, an internal attributional style and need for social approval, as well as family conflict, to predict hopelessness.

Predictors of adolescent hopelessness as a dimensional construct have also been studied in community samples. Studies in disadvantaged samples have suggested that hopelessness is related to family dysfunction and weak social support networks, as well as to exposure to traumatic events (Bolland et al., 2005; Duyan, 2016; Perez-Smith et al., 2002). A longitudinal study of an at-risk sample found that maternal parenting style predicted development of cognitive vulnerabilities for hopelessness, which showed an interaction with negative life events in predicting development of hopelessness (Garber & Flynn, 2001).

The reviewed literature indicates that difficulties in the parent–adolescent relationship may be related to hopelessness in adolescents. In addition to mediation by development of cognitive vulnerabilities, as suggested by the work of Garber and Flynn (2001), difficulties in the parent–adolescent relationship could increase the risk of hopelessness through other pathways as well. Parent–adolescent relationships are obligatory, not voluntary relationships (Laursen & Collins, 2009), and distressing aspects of the relationship may hence be experienced as inescapable. The ability to mentally represent increasingly concrete long-term goals for an adult life also develops in adolescence, and the transitional process to adulthood make these goals highly salient (Nurmi, 1991). At the same time, the adolescent is still dependent on practical and emotional support from their caregivers to be able to approach these goals (Laursen & Collins, 2009). This makes adolescence a developmental period where a positive future is more acutely felt to be dependent on supportive close relationships.

### Parent–adolescent conflict and discrepancies in reporting

Conflict between parents and adolescents is common and to some extent normative (Laursen & Collins, 2009), but the form of conflict resolution achieved and the conflict behaviours that parents and adolescents engage in are systematically related to adolescent adjustment. Repetitive conflict interactions that lead to withdrawal rather than resolution, lack of negotiation and aggressive conflict tactics place adolescents at risk (Branje et al., 2009). Between depressed adolescents and their parents, such dysfunctional forms of conflict are more frequent (Bodner et al., 2018; Sheeber et al., 2007), and a negative predictor of depressive disorder course as well as treatment outcome (Alaie et al., 2020; Asarnow et al., 2009; Feeny et al., 2009).

Discrepancies between the reports of different informants are common when measuring the level of parent–adolescent conflict, as is usual in multi-informant assessment (De Los Reyes et al., 2013). Such discrepancies can be due to differences in access to information about what is reported on, and merely indicate that the phenomenon varies across the contexts in which the informants observe or experience it. This is not likely to be the case with parent–adolescent conflict, where the context is necessarily shared between informants, and neither parents nor adolescents can be regarded as an informant with access to objective information (De Los Reyes et al., 2013). Rather, their reports reflect their individual representation of the conflict state of the relationship, which is built on how they have perceived and interpreted previous conflict situations and how these have been distributed in time (Adams & Laursen, 2001). Informant discrepancies in reports of conflict may therefore represent information about something subtly different than conflict itself, by indicating to what extent the parent–adolescent dyad lack a shared representation of the current state of their relationship. A number of studies have found informant discrepancies to be related to adjustment in children and adolescents (e.g. Nelemans et al., 2016; Ohannessian et al., 2016; Van Heel et al., 2019), supporting the study of informant discrepancies as a variable in itself.

### The present study

Becker-Weidman et al. (2009) noted how adolescent report of conflict, but not parental report, was associated with hopelessness in their sample. Arguing for further research on the specific role of family conflict in development and maintenance of hopelessness, they also recommended investigating how other family members share the adolescents’ perception of their family. Conflict that is unacknowledged by parents may appear unresolvable to the adolescent, giving an expectation of uncontrollable, persistent stress and lack of social support, leading to hopelessness. Discrepancies in report of conflict is one way of operationalising this relational state between adolescents and their parents. This led us to hypothesize that discrepancy between adolescent and parent report of conflict would predict hopelessness among depressed adolescents more than the absolute level of conflict. To evaluate this hypothesis, we compare models predicting hopelessness from the conflict reports of single or multiple informants to a model predicting hopelessness from the level of informant discrepancy.

## Methods

### Participants

We collected data as part of baseline assessments for a randomized controlled trial (clinicaltrials.gov identifier NCT01830088). Participating families were recruited among adolescents referred to two Child and Adolescent Mental Health Services (CAMHS) in South-eastern Norway. During pre-specified recruitment periods, referral letters for adolescents (13–17 years) were examined for mentions of depression or core depressive symptoms (depressed mood, anhedonia or fatigue). The CAMHS routinely administered the Youth Self Report (Achenbach, 1991), and these were screened for raw scores on the Affective Problems subscale above 6 to find depressed adolescents not identified as such in their referral letters (Eimecke et al., 2011). Eligible adolescents or their parents, depending on adolescent age, were contacted by telephone and invited to participate in a randomized trial of family therapy for adolescent depression. About 276 patients were contacted. Participants were required to be currently living with an adult who had become a caregiver for them before age 4, and willing to have this adult participate in treatment. Interested adolescents meeting these criteria were screened with Beck Depression Inventory-II (Beck et al., 1996) over telephone and invited for an assessment session if they scored above 17, a threshold expected to maximize sensitivity (Dolle et al., 2012). This assessment session also served as the intake session to the clinic. One hundred and sixty of the 276 contacted were screened with the BDI-II and 100 of these met with study personnel for a clinical assessment. Adolescents were included in the study if they scored above 15 on the Grid Hamilton Depression Rating scale (GRID-HAMD, Williams et al., 2008) and met Diagnostic and Statistical Manual of Mental Disorders (DSM-IV, American Psychiatric Association, 2000) criteria for a current major depressive episode assessed with the Schedule for Affective Disorders and Schizophrenia for School-age Children – Present and Lifetime Version (K-SADS-PL, Kaufman et al., 1997). Exclusion criteria were psychotic disorders, eating disorders, bipolar disorder, intellectual disability or pervasive developmental disorders. One family withdrew consent after assessment. In all 60 adolescents were included (52 female, 8 male), with 43 fathers and 57 mothers participating. None of the adolescents were in concurrent psychosocial treatment.

### Procedures

Eligible adolescents and their parents met with a study-affiliated clinical psychologist at the CAMHS for an assessment. Parents and adolescents aged 16 or more gave written informed consent to be included in the study, and adolescents below age 16 gave their assent to be included. Adolescents and parents were then interviewed separately. All interviews were video-recorded. Both parents and adolescents completed self-report measures during the appointment.

### Ethical approval

All procedures performed were in accordance with the 1964 Helsinki declaration and its later amendments. The study protocol, participant information letters and consent forms were reviewed and approved by the Regional Committee for Medical and Health Research Ethics for Eastern Norway (REK Øst).

### Measures

#### Hopelessness

There is support for hopelessness as a unidimensional construct that can reliably be measured with very few items (Aish & Wasserman, 2001). We operationalized adolescent hopelessness as the clinician rated hopelessness item in the follow-up interview for Depressive disorders in the K-SADS, scored after interviewing both the adolescent and the parents (Kaufman et al., 1997). This item is scored from 1 to 3, with 1 indicating the absence of hopelessness, 2 indicating a subclinical degree of hopelessness and 3 indicating a clinical degree of depressive hopelessness. The interrater reliability of the scores was assessed by an independent, blinded clinician re-scoring of a random sample of 20 interviews. We estimated the intraclass correlation coefficient for the Hopelessness item following the Bayesian approach of Gajewski et al. (2007). The posterior mean intraclass correlation coefficient was 0.79 (66% and 90% Highest Density Intervals 0.72–0.96 and 0.63–1)^1^ indicating acceptable reliability. Computational details are in the Supplemental Material.

#### Parent–adolescent conflict

Parent–adolescent conflict was assessed with the Perception of the Dyad subscale of the Conflict Behaviour Questionnaire (CBQ, Prinz et al., 1979). The CBQ has seen wide use as a multi-informant measure of parent–adolescent conflict among depressed adolescents (e. g. Curry et al., 2006; Sheeber et al., 2007), and the Perception of the Dyad subscale was among the candidate predictors of hopelessness investigated by Becker-Weidman et al. (2009). Items were translated to Norwegian, and the original author approved a blind reverse translation. Parents completed the measure for their relationship to the adolescent, and the adolescent completed the measure separately for each participating parent.

### Analysis plan

All modelling was conducted in the programming language Stan with the RStan interface (version 2.19.2, Stan Development Team, 2019a) for R (version 3.6.1, R Core Team, 2019). Stan allows for Bayesian inference with estimation by Hamiltonian Monte Carlo (HMC), works well with high-dimensional models, and has sensitive diagnostics for biased or unreliable estimation. The results of a Bayesian analysis are probability distributions of model parameter values, conditional on the observed data and the model with its assumptions. In contrast with classical null-hypothesis testing, Bayesian analysis allows for valid probability statements about the parameter falling within some range (Kruschke, 2018). For the reader unfamiliar with Bayesian statistical approaches, Baldwin and Larson (2017) provide a highly accessible introduction to their use in clinical psychology.

#### Modelling informant discrepancies

**Figure 1.**
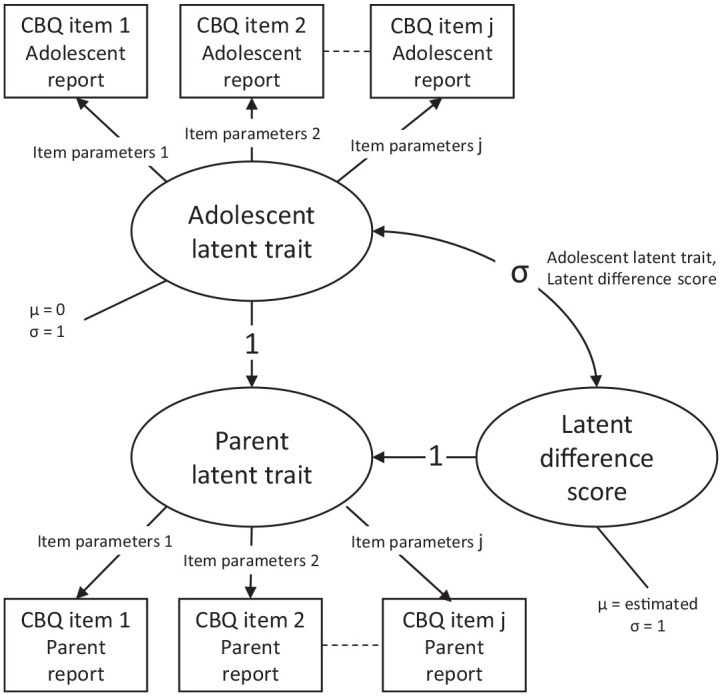
Graphical representation of the latent difference score model.

Informant discrepancies can be modelled using latent difference scores (de Haan et al., 2018). Latent difference scores are obtained by fitting a latent variable model to the responses of both informants and constraining the latent trait variable of one informant to be equal to the sum of a freely estimated latent difference variable and the latent trait variable of the other informant. We implemented this by specifying a two-parameter logistic item response model (IRT model) to the four sets of parent and adolescent responses to the Perception of the Dyad subscale of the CBQ. In the IRT model we specified the latent trait parameter of each parent to be equal to the sum of the latent trait parameter of the adolescent reporting about that parent and a latent difference score parameter. The latent traits of the adolescent and the latent difference scores were specified to have a bivariate normal distribution, with variances constrained to 1, the latent trait mean constrained to 0, and the mean of the latent difference scores and the covariance as estimated parameters. Figure 1 illustrates the structure of the latent difference score model.

Latent difference score modelling assumes measurement invariance across the kinds of respondents whose discrepant reports are to be quantified (de Haan et al., 2018). We evaluated whether this assumption was satisfied following the procedure outlined by Verhagen and Fox (2013). A detailed description of this part of the analysis is available in the Supplemental Material. There was no missing data on the hopelessness variable, but some (0.3%) missing responses to single items on the CBQ. In those cases, we estimated the latent variable based on the observed items. No respondent had more than two single items missing.

#### Modelling predictors of hopelessness and evaluating model fit

To estimate the associations between the latent conflict traits or latent difference scores and the ordinal hopelessness variable, we used ordinal probit regression (Kruschke, 2015). We specified four different models, regressing the ordinal distribution of hopelessness scores on the estimated latent trait of the adolescent (adolescent report), the estimated latent trait of the parent (parent report), both latent traits (multi-informant report), and the estimated latent difference scores (informant discrepancy). We fitted each of these four models simultaneously to both the mother–adolescent data and the father–adolescent data, in order to share item parameters for the IRT models. We compared these four models using approximate leave-one-out cross-validation (PSIS-LOO), which yields estimates of expected out-of-sample predictive accuracy, similarly to information criteria such as the AIC, but with the advantage of sensitive diagnostics for when estimates are unreliable (Vehtari et al., 2017). Estimates of the leave-one-out predictive density from PSIS-LOO can also be used for stacking of predictive distributions (Yao et al., 2018). This is a procedure that given a set of models finds the weighted combination of models that has a predictive distribution that is closest to the data generating process, allowing model weights to be interpreted as the contribution to predictive accuracy gained from each model if these were combined as one single model (Yao et al., 2018).

#### Prior distributions, estimation and validation of convergence

In Bayesian data analysis, prior distributions must be specified for all model parameters, representing our assumptions and knowledge about the parameters irrespective of the data (Gelman et al., 2013). The weakly informative prior distributions used in this analysis and the reasoning behind choosing them can be found in the Supplemental Material, and we encourage the reader to review them and consider whether they are reasonable.

We estimated all models running four Markov chains with the standard algorithm, with 1,000 warmup iterations and drawing 2,500 samples from each chain (Stan Development Team, 2019b). Rubin-Gelman statistics were below 1.01 for all parameters, and there were no negative Stan convergence diagnostics, indicating valid sampling from the posterior distribution.

## Results

### Sample characteristics and latent variable distributions

The distributions of K-SADS Hopelessness scores (1/2/3) were 11/17/23 for adolescents with mothers reporting and 10/11/21 for the adolescents with fathers reporting. The posterior means of the latent trait for adolescent report of conflict had a range of −1.48 to 2.53 for father–adolescent conflict and −1.39 to 1.53 for mother–adolescent conflict. The posterior means of the latent difference scores had a range of −1.42 to 1.56 for father–adolescent conflict, and −1.75 to 1.73 for mother–adolescent conflict. The correlation of the latent difference scores and the latent traits had a posterior mean of −0.34 (*SD* 0.19) for father–adolescent conflict and a posterior mean of −0.33 (*SD* 0.19) for mother–adolescent conflict.

### Cross-validation and stacking of models

We then used the R-package loo (Vehtari et al., 2019), to compare models using PSIS-LOO. We also calculated model stacking weights (Yao et al., 2018). Results are displayed in Table 1.

**Table 1.** Comparison of models with PSIS-LOO and stacking.

Model	Difference (*SE*)	P-loo (*SE*)	Stacking weight
Conflict with mother			
Informant discrepancy	–	4.5 (0.4)	0.88
Adolescent report	–0.5 (1.2)	3.4 (0.4)	0.12
Parent report	–0.6 (0.9)	3.6 (0.4)	0
Multi-informant report	–1.1 (0.3)	5.7 (0.6)	0
Conflict with father			
Informant discrepancy	–	6.1 (0.6)	0.85
Adolescent report	–0.8 (1.6)	3.5 (0.4)	0.15
Parent report	–0.8 (1.5)	3.8 (0.5)	0
Multi-informant report	–1.1 (0.3)	7.2 (0.8)	0

*Note*. Difference (*SE*) = difference to model with highest estimated predictive accuracy, higher (less negative) numbers indicate better fit; P-loo (*SE*) = effective number of parameters, an estimate of model complexity; weight = model weight from the stacking procedure.

For both the mother–adolescent and the father–adolescent data, the models with latent difference scores as the only independent variable are better than the models with the reports of multiple informants. The differences in predictive accuracy to the models with single informants is within the standard error of the estimate. The stacking procedure does however give most weight to the model with latent difference scores as the only independent variable, for both mothers and fathers, and some weight to the models with adolescent report as the independent variable. This means that once informant discrepancies are taken into account, there is not much predictive accuracy to gain from information about the absolute level of conflict reported by the adolescent, and when both of these are taken into account, there is nothing to gain from the information reported by the parents.

### Regression model parameter estimates and model predictive distributions

The parameter estimates of the fitted regression models are summarised in Table 2. The full sets of samples drawn from the posterior distributions of all four models, and the Stan model code are available at 10.17605/OSF.IO/75ZER.

**Table 2.** Regression parameter estimates from informant discrepancy and adolescent report models.

Parameters	Mean	*SD*	Median	66% HDI	90% HDI	ESS
Informant discrepancy – mother						
Regression coefficient	–0.25	0.23	–0.24	–0.43; –0.01	–0.60; 0.13	9,087
First cutpoint	–0.88	0.20	–0.87	–1.07; –0.70	–1.19; –0.55	16,555
Second cutpoint	0.05	0.17	0.05	–0.12; 0.21	–0.25; 0.32	11,270
Adolescent report – mother						
Regression coefficient	0.08	0.19	0.08	–0.10; 0.25	–0.23; 0.38	13,228
First cutpoint	–0.87	0.20	–0.86	–1.04; –0.66	–1.19; –0.55	14,336
Second cutpoint	0.03	0.17	0.03	–0.13; 0.20	–0.26; 0.31	13,092
Informant discrepancy – father						
Regression coefficient	–0.42	0.33	–0.4	–0.68; –0.08	–0.96; 0.10	7,243
First cutpoint	–0.73	0.23	–0.73	–0.95; –0.51	–1.12; –0.37	15,468
Second cutpoint	0.13	0.21	0.12	–0.08; 0.32	–0.22; 0.48	11,508
Adolescent report – father						
Regression coefficient	0.06	0.19	0.06	–0.13; 0.23	–0.25; 0.37	12,709
First cutpoint	–0.76	0.21	–0.75	–0.93; –0.53	–1.09; –0.4	15,386
Second cutpoint	0.04	0.19	0.04	–0.14; 0.22	–0.26; 0.37	13,631

*Note*. Mean = posterior mean; *SD* = posterior standard deviation; median = posterior median; 66% and 90% HDI = the 66% or 90% highest density interval; ESS = Effective sample size, the estimated number of effectively independent draws from the posterior distribution.

The 90% and 66% HDIs are quite wide, showing the considerable uncertainty in the estimates. If we reason that a standardised regression coefficient between −0.1 and 0.1 is practically close enough to 0 to be of little theoretical interest in this case, we can use the posterior distribution to calculate the probability of a regression coefficient that is negative and of a larger magnitude than −0.1 (Kruschke, 2018). For the model with informant discrepancies these probabilities are .74 for mother–adolescent conflict and .85 for father–adolescent conflict. There seems to be a difference in the magnitude of the association between mothers and fathers, but the posterior distributions of the regression coefficients overlap considerably. The mean posterior difference between the mother–adolescent regression coefficient and the father–adolescent regression coefficient is 0.18, with a standard deviation of 0.40. The probability of the father–adolescent regression coefficient having a larger negative magnitude than the mother–adolescent regression coefficient is 0.67 – probable, but far from certain. A negative regression coefficient in this case signifies that hopelessness is expected to increase when parents report less conflict than their adolescents.

Visualisation of the predictive distribution of the model can be helpful to understand the implications of a fitted model. Choosing some values for the independent variables, we can make repeated draws from the predicted distributions of the dependent variable at those levels of the independent variable, with the drawn distributions of dependent variable values containing the uncertainty of the model fit.

**Figure 2.**
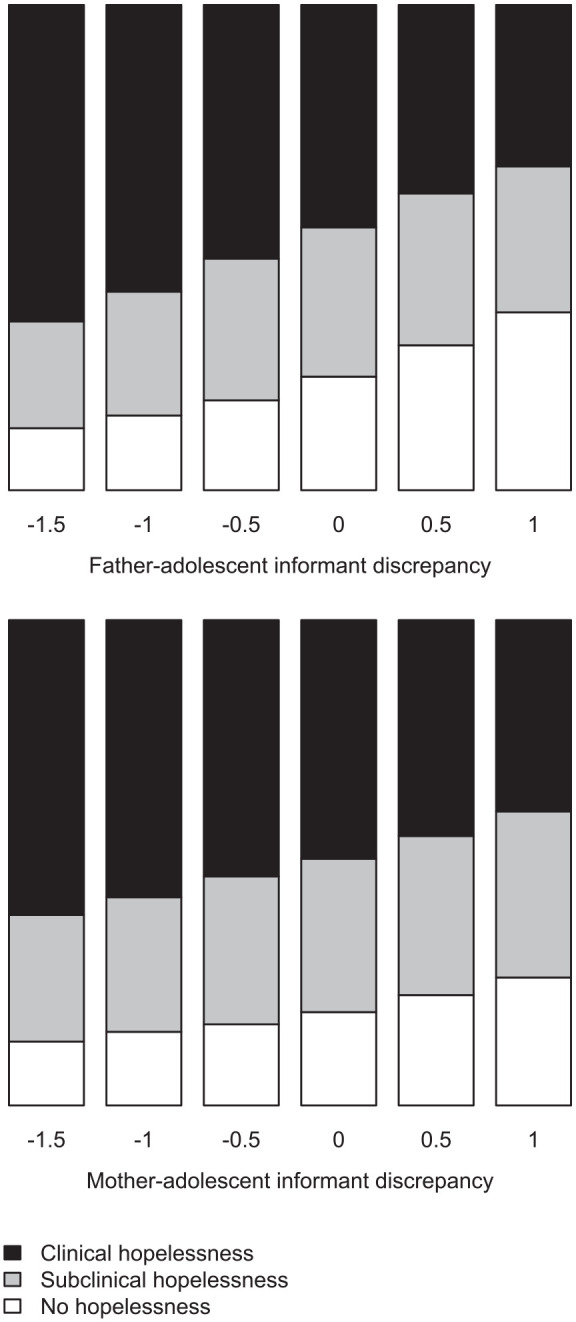
Plots of model predictions across the range of informant discrepancy.

In Figure 2, we have plotted the distributions of hopelessness values predicted by the stacked models (combining draws according to the stacking weights) for both father–adolescent conflict and mother–adolescent conflict, at different levels of informant discrepancy, holding adolescent report of conflict constant at the mean.

With the uncertainty in the model fit preserved in the plotted distributions, the main weight of the evidence is still on the frequency of clinically significant hopelessness increasing when parents report less conflict than the adolescent, in particular for father–adolescent informant discrepancy. To check that the low number of male adolescents did not conceal gender-specific associations, we refitted all models with the male adolescents held out, and compared model fit and parameter estimates to models fitted to the full sample. Removing male adolescents did not improve fit as assessed by PSIS-LOO, and parameter estimates were highly similar.

## Discussion

Given the clinical importance of hopelessness in adolescent depression, it is unfortunate that predictors of adolescent hopelessness have received relatively little research attention. In this study we found preliminary support for our hypothesis that parent–adolescent informant discrepancy in report of conflict would be associated with hopelessness among depressed adolescents. The present findings suggest that informant discrepancies capture information about some way parent–adolescent dyads differ that is distinct from the level of conflict, and which is related to adolescent hopelessness.

We found evidence for a relationship between hopelessness and parents reporting less conflict than the adolescent. The same pattern of discrepancy, but concerning family routines and chaos rather than conflict, was found to predict development of depressive symptoms in a longitudinal study of a community sample (Human et al., 2016). Although speculative at present, it is possible that informant discrepancies, across different variables, all indicate similar negative family processes. Such family processes could be difficult to assess accurately using self-report, in which case informant discrepancies would have potential for clinical assessment. Further research is needed to evaluate whether there are common family processes that predict informant discrepancies, and for what classes of variables. As noted by De Los Reyes et al. (2013), the meaning of informant discrepancies will differ when what is reported on is part of a context shared by the informants, and when it is not. It is likely that discrepancies due to different access to information is less indicative of negative family processes than mismatched perceptions of shared contexts like family routines, family chaos and parent–adolescent conflict.

Another recent longitudinal study of a community sample found discrepancy in reports of negative interactions between fathers and adolescents, but not mothers, to predict development of depressive symptoms (Nelemans et al., 2016). This is in line with our finding that the association might be stronger for the father–adolescent relationship.

Unlike Becker-Weidman et al. (2009), we did not find that adolescent report of conflict was strongly associated with hopelessness in itself. However, there are several differences in statistical analysis and measurement methodology between these studies that make direct comparisons difficult.

### Limitations

This study is limited by a small sample size, which is reflected in the uncertainty of the posterior estimates. Applying Bayesian data analysis is an advantage in such cases, as the uncertainty is preserved and visible in the results, and inference does not rely on asymptotic properties of the sampling distribution. Careful attention to choice of priors and validation of convergence is crucial in such cases (McNeish, 2016), and this has been observed in the present analysis. Considering the sample size and the uncertainty of the posterior, we view the present findings as an interesting lead, deserving attempted replication. Replication in a sample with a larger proportion of male adolescents would help clarify whether such an association is gender specific, as the number of male adolescents in this sample was low. We found no evidence of a gender-specific association but can clearly not rule it out. The cross-sectional design also limits the causal inferences that may be drawn. It cannot be ruled out that hopelessness is related to adolescents overestimating the level of conflict relative to the parents (i.e. depressive distortion, De Los Reyes et al., 2013), although the low probability of a strong positive association between adolescent report of conflict and hopelessness does make this interpretation less reasonable.

## Conclusion

That informant discrepancies appear to have a stronger association with hopelessness than adolescent report alone, is a reminder of why multi-informant assessments are vital in the study of relational phenomena. Relying on a single informant or on analysing the reports of multiple informants separately can probably conceal or misrepresent associations, as would have been the case if we had only analysed adolescent report of conflict. If replication should support these preliminary findings, it would imply that clinicians working with depressed adolescents and their families need to be attentive not only to conflict in the family, but also to differences in the perception of conflict. When adolescents find parent–adolescent conflict more severe than their parents, it seems to indicate different family processes than heightened conflict alone, and this may have implications for intervention.

These results further demonstrates how latent difference scores (de Haan et al., 2018) can be combined with item response theory for studying informant discrepancies, and how Stan (Stan Development Team, 2019b) is a powerful and flexible computational framework for such analyses. Change in informant discrepancy should also be considered for inclusion as a mediator variable treatment studies, in particular those involving family-oriented interventions.

## Supplemental Material

calculation_of__icc – Supplemental material for Using informant discrepancies in report of parent–adolescent conflict to predict hopelessness in adolescent depression Click here for additional data file. Supplemental material, calculation_of__icc for Using informant discrepancies in report of parent–adolescent conflict to predict hopelessness in adolescent depression by Erling W Rognli, Marianne Aalberg and Nikolai Olavi Czajkowski in Clinical Child Psychology and Psychiatry

measurement_invariance – Supplemental material for Using informant discrepancies in report of parent–adolescent conflict to predict hopelessness in adolescent depression Click here for additional data file. Supplemental material, measurement_invariance for Using informant discrepancies in report of parent–adolescent conflict to predict hopelessness in adolescent depression by Erling W Rognli, Marianne Aalberg and Nikolai Olavi Czajkowski in Clinical Child Psychology and Psychiatry

priors – Supplemental material for Using informant discrepancies in report of parent–adolescent conflict to predict hopelessness in adolescent depression Click here for additional data file. Supplemental material, priors for Using informant discrepancies in report of parent–adolescent conflict to predict hopelessness in adolescent depression by Erling W Rognli, Marianne Aalberg and Nikolai Olavi Czajkowski in Clinical Child Psychology and Psychiatry

## Notes

1. We generally choose to report the 66% and 90% Highest Density Intervals (see Kruschke, 2018), with the suggested interpretation of the likely and very likely range within which the true parameter value lies (Mastrandrea et al., 2010).
