# Supplementary material for: Using informant discrepancies in report of parent–adolescent conflict to predict hopelessness in adolescent depression
Source: Clin Child Psychol Psychiatry. 2020 Nov 5;26(1):96–109. doi: 10.1177/1359104520969761 (PMC7802054; doi:10.1177/1359104520969761)
Supplement: calculation_of__icc – Supplemental material for Using informant discrepancies in report of parent–adolescent conflict to predict hopelessness in adolescent depression [file calculation_of__icc.pdf]

The Bayesian approach to calculating Intraclass Correlation Coefficients (ICCs) described in the work of Gajweski and colleagues (2007) concern pooling information across ratings at different sites, which was not relevant for our application. However, they develop the idea of using a hierarchical ordinal probit model, with ratings by different raters nested within rated subjects. The observed ratings are taken to indicate a latent normal variable, defined as the sum of subject effects and rater errors. The posterior distribution of the variances of the subject effects and rater errors can then be used to calculate the posterior distribution of the two-way, random effects, single measures ICC (McGraw & Wong, 1996). An advantage of this approach is that it avoids treating ordinal ratings as continuous variables.

The following Stan program implements this approach with a non-centered parametrization. We have specified half-t distributions on the variances, with the degrees of freedom parameter estimated, to allow for heavy-tailed distributions of errors. The gamma (2, 0.1) prior on the degrees of freedom allows for anything from a half-cauchy to a practically half-normal distribution. The normal (0, 10) prior on the cutpoints is very weakly informative with a standard normal latent variable.

Gajewski, B. J., Hart, S., Bergquist-Beringer, S., & Dunton, N. (2007). Inter-rater reliability of pressure ulcer staging: ordinal probit Bayesian hierarchical model that allows for uncertain rater response. Statistics in Medicine, 26(25), 4602-4618. doi:10.1002/sim.287

McGraw, K. O., & Wong, S. P. (1996). Forming inferences about some intraclass correlation coefficients. Psychological Methods, 1(1), 30-46. doi:10.1037/1082-989x.1.1.30

```
data{

  int subjects;
  int raters;
  int categories;
  int scores[subjects*raters];
  int subject_index[subjects*raters];
}

parameters{

  real<lower=0> sigma_effects;
  real<lower=0> sigma_errors;
  real<lower=1> nu_effects;
  real<lower=1> nu_errors;
  vector[subjects] effects_raw;
  vector[subjects*raters] errors_raw;
  ordered[categories-1] cutp;
}

model{

  vector[subjects] effects = effects_raw * sigma_effects;
  vector[subjects*raters] errors = errors_raw * sigma_errors;
  cutp ~ normal(0,10);

  effects_raw ~ std_normal();
  nu_effects ~ gamma(2,0.1);
  sigma_effects ~ student_t(nu_effects,0,1);

  errors_raw ~ std_normal();
  nu_errors ~ gamma(2,0.1);
  sigma_errors ~ student_t(nu_errors,0,1);

  scores ~ ordered_probit(effects[subject_index] + errors, cutp);
}

generated quantities{

  real<lower=0, upper=1> icc = sigma_effects/(sigma_effects + sigma_errors);
}
```
