# Supplementary material for: Using informant discrepancies in report of parent–adolescent conflict to predict hopelessness in adolescent depression
Source: Clin Child Psychol Psychiatry. 2020 Nov 5;26(1):96–109. doi: 10.1177/1359104520969761 (PMC7802054; doi:10.1177/1359104520969761)
Supplement: measurement_invariance – Supplemental material for Using informant discrepancies in report of parent–adolescent conflict to predict hopelessness in adolescent depression [file measurement_invariance.pdf]

Latent discrepancy score modelling assumes measurement invariance across the kinds of respondents whose discrepant reports are to be quantified (de Haan, Prinzie, Sentse, & Jongerling, 2018). The latent trait estimates from an IRT model has measurement invariance across different kinds of respondents if the model fits equally well to all of them (de Ayala, 2009). To evaluate whether the assumption of measurement invariance was satisfied, we fitted models with item parameters freely estimated for mothers, fathers, and for adolescent report on mother and father separately, and compared these to the fit of models with item parameters constrained to be equal for all four kinds of respondent, following the procedure described by Verhagen and Fox (2013). To compare these models we used approximate leave-one-out cross-validation (PSIS-LOO, Vehtari, Gelman, & Gabry, 2017). This a computationally efficient way of estimating the fit of a model to future data, similarly to the Deviance Information Criterion used by Verhagen and Fox (2013), but which also has the advantage of sensitive diagnostics for when estimates are likely to be biased (Vehtari et al., 2017). Item-wise PSIS-LOO showed that for all but two items, the models with item parameters constrained to be equal were estimated to have better out-of-sample fit for all four kinds of respondent. The items fitted better by separate item parameters were these, reverse scored: “2: We do a lot of things together.” and “4: I enjoy the talks we have.” We therefore omitted these two items when estimating the latent difference scores. Visual inspection of the posterior distributions of item characteristic curves plotted against the data indicated good fit for the remaining items. These plotted curves are reproduced on the next page.

de Ayala, R. J. (2009). The theory and practice of item response theory. New York: Guilford Press.

de Haan, A., Prinzie, P., Sentse, M., & Jongerling, J. (2018). Latent difference score modeling: A flexible approach for studying informant discrepancies. Psychological Assessment, 30(3), 358-369. doi:10.1037/pas0000480

Vehtari, A., Gelman, A., & Gabry, J. (2017). Practical Bayesian model evaluation using leave-one-out cross-validation and WAIC. Statistics and Computing, 27(5), 1413–1432. doi:10.1007/s11222-016-9696-4

Verhagen, A. J., & Fox, J. P. (2013). Bayesian tests of measurement invariance. British Journal of Mathematical and Statistical Psychology, 66(3), 383-401. doi:10.1111/j.2044-8317.2012.02059.x

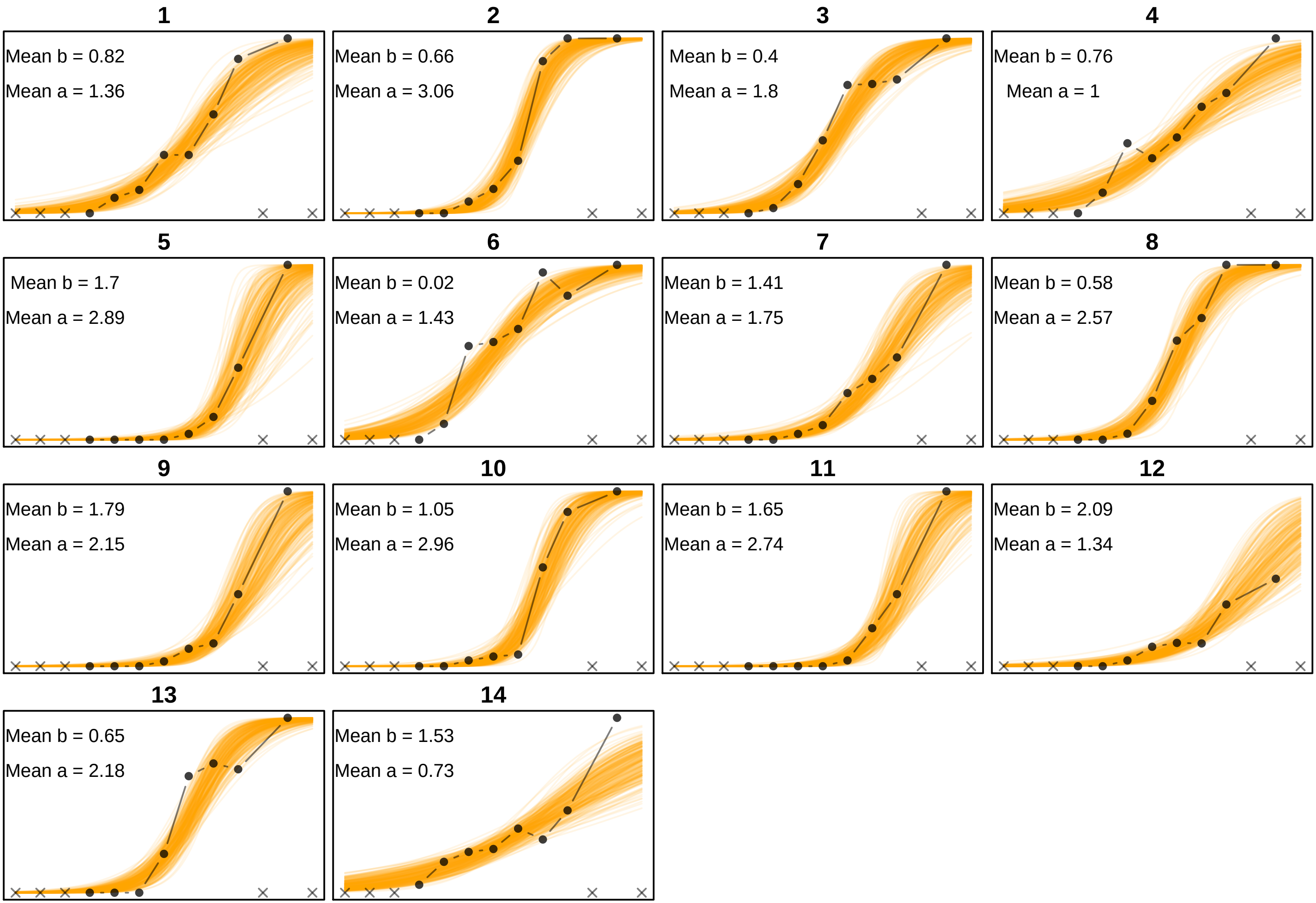
1
Mean b = 0.82
Mean a = 1.36
2
Mean b = 0.66
Mean a = 3.06
3
Mean b = 0.4
Mean a = 1.8
4
Mean b = 0.76
Mean a = 1
5
Mean b = 1.7
Mean a = 2.89
6
Mean b = 0.02
Mean a = 1.43
7
Mean b = 1.41
Mean a = 1.75
8
Mean b = 0.58
Mean a = 2.57
9
Mean b = 1.79
Mean a = 2.15
10
Mean b = 1.05
Mean a = 2.96
11
Mean b = 1.65
Mean a = 2.74
12
Mean b = 2.09
Mean a = 1.34
13
Mean b = 0.65
Mean a = 2.18
14
Mean b = 1.53
Mean a = 0.73
