# Supplementary material for: Using informant discrepancies in report of parent–adolescent conflict to predict hopelessness in adolescent depression
Source: Clin Child Psychol Psychiatry. 2020 Nov 5;26(1):96–109. doi: 10.1177/1359104520969761 (PMC7802054; doi:10.1177/1359104520969761)
Supplement: priors – Supplemental material for Using informant discrepancies in report of parent–adolescent conflict to predict hopelessness in adolescent depression [file priors.pdf]

Prior Distributions and Reasoning for Choices of Prior

Parameter(s)	Prior Distribution	Reasoning
Regression coefficients	Normal (0, 1)	Weakly informative, given that the latent independent variables are standardized and that large regression coefficients would be very surprising.
Thresholds for probit regression	Normal (0, 3)	Weakly informative, given an approximately standard normal latent variable and two thresholds.
Latent traits and latent difference scores	Multivariate normal with location 0 for latent traits, a normal (0,1) hyperprior on the mean of the latent difference scores, both variances constrained to 1, and a LKJ (2) hyperprior on the correlation matrix.	Defines latent trait as a standard normal variable for model identifiability and interpretability, estimates the mean of latent difference scores, and the correlation between latent difference scores and latent traits, and restricts the latent traits and the latent difference scores to have the same scale.
CBQ IRT model item thresholds	Hierarchical normal prior with the hyperpriors Normal (0,3) for location and Half-students' t (3,0,1) for scale.	Hierarchical prior with weakly informative hyperpriors, estimating the distribution of item thresholds from the data. Wide hyperprior on the location of the distribution, as the interdependent latent variables are constrained to standard normal.
CBQ IRT model item discrimination	Gamma (2, 0.5)	Places most of the prior weight on discrimination between about 1 and 5, which is the most probable range for items of an established instrument, but does not rule out higher or lower values.
